# Reliability and validity of the *dutch eating behavior questionnaire* in an online format for university students from low-income regions in a pandemic context: A 24 hour MESYN study

**DOI:** 10.3389/fepid.2022.1036631

**Published:** 2023-01-11

**Authors:** Millena Vaz de Carvalho, Antonio Gibran de Almeida Cardoso, Shirley Cunha Feuerstein, Rosana Rodrigues de Sousa, Tatiana Sadalla Collese, Francisco Leonardo Torres-Leal, Marcus Vinicius Nascimento-Ferreira, Augusto Cesar Ferreira De Moraes

**Affiliations:** ^1^ Health, Physical Activity and Behavior Research (HEALTHY-BRA) Group, Universidade Federal do Tocantins, Miracema do Tocantins, Brazil; ^2^ Instituto de Ensino Superior do Sul do Maranhão (IESMA/UNISULMA), Imperatriz, Brazil; ^3^ Metabolic Diseases, Exercise and Nutrition Research Group (DOMEN), Department of Biophysics and Physiology, Centre for Health Sciences, Federal University of Piaui, Teresina, Brazil; ^4^ School of Public Health, University of São Paulo, São Paulo, Brazil; ^5^ São Camilo University Center, São Paulo, São Paulo, Brazil; ^6^ Youth/Child Cardiovascular Risk and Environmental (YCARE) Research Group, Faculdade de Medicina, Universidade de Sao Paulo, São Paulo, Brazil; ^7^ The University of Texas Health Science Center at Houston, School of Public Health Austin Campus, Department of Epidemiology, Human Genetics, and Environmental Science, Michael & Susan Dell Center for Healthy Living, Austin, TX, United States

**Keywords:** questionnaire, emotional eating, restrained eating, external eating, psychometric properties, adults, reliability study

## Abstract

**Objective:**

To test the reliability and validity of the *Dutch Eating Behavior Questionnaire* (DEBQ) in an online format in university students from low-income regions.

**Methods:**

We applied the questionnaire to a sample of 195 and 117 university students from a low-income region (Gini index of 0.56) to study validity and reliability, respectively. The DEBQ consists of 33 items on eating behavior in three dimensions/factors: emotional eating, restrained eating and external eating. The questionnaire was administered twice at 2-week intervals. We tested the reliability *via* temporal stability and internal consistency and construct validity *via* exploratory and confirmatory factor analysis.

**Results:**

For reliability, we identified an acceptable Spearman correlation coefficient (rho > 0.30 and *p* < 0.05) and Cronbach's alpha (*α* ≥ 0.70) for all DEBQ items. In the exploratory analysis, we identified 6 factors representing a mix of original and additional factors, with an explained variance of 69.1%. In the confirmatory analysis with structural equation modeling, we observed better global model adjustment for the 6-factor model with the Tucker–Lewis index and comparative fit index closer to one, as well as root mean square error of approximation closer to zero than the original (3-factor) model. Using generalized structural equation modeling, we also observed a better fit in latent class modeling for the 6-factor model (AIC: 16990.67; BIC. 17874.38) than for the 3-factor model (AIC: 17904.09; BIC: 18342.67).

**Conclusion:**

The online format of the DEBQ has acceptable reliability and validity for measuring eating behavior in university students from low-income regions.

## Introduction

Eating behaviors are food choices moderated by consumption trends, personal preferences, specific diets and calorie count (1) and constitute a complex construct that can be influenced by several variables (2). In the university environment, eating behavior seems to be associated with individual factors (e.g., knowledge and perceptions about food), social factors (e.g., peer influence and social norms), environmental factors (e.g., food availability and prices) and academic factors (e.g., campus culture and frequency of academic exams) (3).

The COVID-19 pandemic imposed many restrictive measures aimed at reducing the impact of this disease, and as a result, lifestyles were drastically altered, affecting eating behaviors (2). Epidemiological studies have also been affected, leading to an explosion of nonface-to-face research (4) and tools for the online environment (5). Among the instruments used for the evaluation of eating behavior, the *Dutch Eating Behavior Questionnaire* (DEBQ) has a consistent psychometric capacity with a robust structure, which provides reliable and valid information in different populations (including adults from different cultures) (6–10).

Although the adoption of online tools may have reduced economic and logistical costs compared to face-to-face data collection and provided greater potential engagement (5), some methodological challenges should be considered (4, 5, 11). The level of education of the participants and access to the internet may distort recruitment and the ability to respond to online tools, especially in populations with marked socioeconomic and educational differences (4). Thus, although DEBQ is a valid tool for measuring eating behavior (face-to-face and online format), to the best of our knowledge, it has not yet been tested online in low-income regions. In addition, the construct has not been tested in a pandemic context. Considering the assumption that contexts (e.g., different countries) and subgroups can expose the latent factors of a construct, especially in respondents exposed to rapidly changing environments (4) such as that observed in the COVID-19 pandemic, we hypothesized that the DEBQ, in an online format, has acceptable reliability and validity in university students from low-income regions in a pandemic context.

## Methods

### Study design

This study assessed psychometric properties of the DEBQ: reliability (temporal stability and internal consistency) and validity (construct/structural) (12). The study is part of a multicenter observational longitudinal project entitled 24 hour Movement Behavior and Metabolic Syndrome (24 h-MESYN). The data collection of this study occurred between February and June 2021 in a single research center. Detailed information about the 24 h-MESYN study can be found elsewhere (13).

### Ethical aspects

The study followed the ethical principles for research with human beings: i) Declaration of Helsinki, revised in 2008, Seoul, Korea; ii) resolution of CNS 466/12; iii) guidelines for the conduct of research activity during the COVID-19 pandemic (available at https://prp.usp.br/wp-content/uploads/sites/649/2016/05/prp_covid_2.pdf); and iv) guidelines for research in a virtual environment (OFÍCIO CIRCULAR N° 2/2021/CONEP/SECNS/MS). After being invited and agreeing to participate in the study, only students who freely signed the informed consent form (online) participated in the study. The study approval was given by *Centro Universitário do Maranhão* (UNICEUMA) Human Research Ethics Committee (ID: 4,055,604).

### Population, sample and sampling

The population of this project was composed of students enrolled in a higher education institution selected by convenience. The institution is located in a city in the south of the state of Maranhão (Brazil), which has a Gini index of 0.56 (14). In 2020, our based population was 2,225 students enrolled in nine undergraduate programs: Administration, Law, Physical Education, Nursing, Aesthetics and Cosmetics, Physiotherapy, Nutrition, Psychology and Social Work.

The sample size was calculated according to the assumptions of Nascimento-Ferreira (15). The parameters used to calculate the sample size were *α* of 0.05, *β* of 0.10 (or power of 90%) and Pearson's correlation coefficient of 0.30 (minimum required for a correlation matrix in a study of exploratory factor analysis) (16). Based on these parameters, we estimated a sample of 85 students for the current study. However, as the 24 h-MESYN project was designed to evaluate the psychometric properties of at least five other subjective tools, we established a total of 342 participants to meet minimum aspects (example: 80.0% power) for all tools tested (13).

Next, we conducted a stratified sampling with the following strata: biological sex (at least 60.0% for the female biological sex), age (at least 25.0% for students up to 20 years of age) and study program (at least minus 60.0% in the health area), based on previous cohorts (17, 18). The potential participants were sampled in the university entrance hall and open areas following health recommendations.

### Inclusion and exclusion criteria

We included regularly enrolled students with a minimum age of 18 years who were selected for the study and who signed an informed consent form. We excluded students who did not complete or incorrectly completed the questionnaires. We excluded the students who presented with physical disability or pregnancy from the analysis.

### Harmonization of measures

The entire process of data quality was divided into methodology harmonization and fieldwork training. Regarding methodology harmonization and fieldwork training, the researchers participated in training (lasting 20 h of work) to understand the flow of data collection and to become familiar with the questionnaire (13). The training was offered at the institution by the project coordinator (Nascimento-Ferreira). During training, we also reviewed the online version of the questionnaire (13).

### Procedures

The first step was carried out in person at the institution's premises. In this step, we explained the project and sent the link *via* an instant messaging application (*WhatsApp*) with the informed consent form. In this phase, we carried out the study invitation following national health protocols related to COVID-19 (e.g., wearing face mask, avoiding close contact). In the second step, after signing the online form, the participants answered the questionnaire for the first time (Q1). Here, we sent up to three reminders in the case of electronic questionnaire no response. In the third step, two weeks after the previous step, we resent the link to the same questionnaire, and the participants answered a second time (Q2). The questionnaire was sent to only those who replied to it in Q1. In the latter two steps, our contact was restricted to messaging *via* WhatsApp.

### Study variables

We retrieved all data using subjective tools for biological sex, age, academic course, academic shift and eating behavior.

### Instruments

We evaluated eating behavior using the *Dutch Eating Behavior Questionnaire* (DEBQ) (19), a questionnaire validated in European (6, 7) and Brazilian (20) adults. The 33-item self-report questionnaire is based on three dimensions (or factors): emotional eating (Items 1, 3, 5, 8, 10, 13, 16, 20, 23, 25, 28, 30 and 32), restrained eating (Items 4, 7, 11, 14, 17, 19, 22, 26, 29 and 31) and external eating (Items 2, 6, 9, 12, 15, 18, 21, 24, 27 and 33). The responses were scored on a 5-point Likert scale (never, rarely, sometimes, often and very often). To generate the score of each scale, we first assigned a value from 1 (never) to 5 (very often) for the responses, and then the scores of the items of each dimension were added and the gross value divided by the number of items in the dimension (6). In addition, we retrieved sociodemographic (biological sex and age) and academic (course and academic shift) information. All information was retrieved using an online questionnaire (available at https://forms.gle/L92wXsVaxxfPNgpE8).

### Statistical analysis

We conducted all statistical analyses in Stata software (version 15.0, Stata Corporation, College Station, TX, United States), with a statistical significance criterion of 95.0% (*p* ≤ 0.05). To assess sensitivity, we applied the chi-square goodness-of-fit test. To test reliability, we assessed temporal stability using Spearman's correlation coefficient with cutoff ≥ 0.30 (for acceptable test-retest [Q1 vs. Q2] reliability) (21). In addition, we performed an internal consistency analysis by determining the Cronbach's coefficient alpha for all items and each factor identified in the exploratory analysis using Cronbach's alpha with cutoff ≥ 0.70 (for acceptable internal reliability) (22).

For the validity of the construct, we assessed the questionnaire structure *via* exploratory and confirmatory factor analysis. In the exploratory analysis, we applied exploratory factor analysis with Varimax rotation (16). We extracted the factors based on the *Kaiser* rule, with eigenvalues >1 for factor retention (16). Initially, we performed a preliminary analysis to determine whether the data were feasible with the Kaiser‒Meyer‒Olkin test (KMO > 0.50) for sample adequacy and the Bartlett test (*p* < 0.05 as statistically significant) for sphericity of data (16). In the confirmatory analysis, we employed structural equation modeling (SEM) to test the best factor structure adjustment by comparing the DEBQ original model with the model identified in the exploratory analysis (23, 24), and we employed generalized structural equation modeling (GSEM) to test the best fitting of latent variables (24) (also interpreted as factors or domains). In the SEM, each model was composed of exogenous latent variables and observed exogenous variables in our analysis (24). A latent exogenous variable is determined to be outside the model if paths only originate from it or, equivalently, no path points to it (24), whereas an observed exogenous variable is the observed item reported by the participant (24). Thus, the SEM is more restrictive than the exploratory factor analysis (23). In this sense, all cross loadings were constrained to be zero (23). Thus, to convert the model from exploratory factor analysis to SEM, items loaded into more than one factor were included only in the factor with the greatest factor loading. We compared the models with the likelihood-ratio test (24). The Tucker‒Lewis index (TLI), comparative fit index (CFI), root mean square error of approximation (RMSEA), and standardized root mean squared residual (SRMR) were used to evaluate the quality of the model. The higher TLI and CFI values and lower RMSEA and SRMR values were considered to indicate a better fit (25), and the statistical significance of the likelihood-ratio test was set at *p* ≤ 0.05. In the GSEM, we assessed latent class models comparing endogenous latent variables (a variable determined by the model) (24). In our analysis, we specified the number (up to one more class than observed in the exploratory factor analysis) of latent classes and then estimated the fit (24). We assessed the model fit using information criteria measures (Akaike information criterion [AIC] and Bayesian information criterion [BIC]) (24).

## Results

Of the 342 invited students, we identified a 43.0% prevalence of refusal to participate in the study in Q1, and a 40.0% prevalence in Q2. In this sense, we identified 57.0% of response rate (students who were invited and answered Q1) in the Q1 and 60.0% in the Q2 (students who answered Q1 and Q2). Table 1 shows the description of the sample and sensitivity analysis, according to demographic and academic variables. In both applications, we observed greater participation of female students (Q1 = 74.9% and Q2 = 72.7%), those aged between 21 and 25 years (Q1 = 44.6% and Q2 = 45.7%), those taking a Physical Education academic course (Q1 = 24.0% and Q2 = 24.8%) and those taking night classes (Q1 = 61.3% and Q2 = 62.4%), but with no significant difference that characterized differential bias.

**Table 1:** Sensitivity analysis based on sociodemographic and academic variables.

Variables		Q1 (*n* = 195)	Q2 (*n* = 117)	*p* ^†^
		*n* (%)	*n* (%)	
Biological sex	Male	49 (25.1)	32 (27.4)	0.36
	Female	146 (74.9)	85 (72.7)	
Age	18 to 20 years	46 (23.6)	31 (26.7)	0.63
	21 to 25 years	87 (44.6)	53 (45.7)	
	26 to 30 years	36 (18.5)	17 (14.7)	
	31 to 35 years	14 (7.2)	6 (5.2)	
	36 to 52 years	12 (6.2)	9 (7.8)	
Academic course	Nutrition	15 (7.7)	7 (6.0)	0.17
	Physical Education	47 (24.0)	29 (24.8)	
	Nursing	25 (12.8)	14 (12.0)	
	Aesthetics and Cosmetics	8 (4.1)	2 (1.7)	
	Physiotherapy	34 (17.3)	22 (18.8)	
	Law	19 (9.7)	13 (11.1)	
	Psychology	39 (19.9)	25 (21.4)	
	Social work	5 (2.6)	3 (2.56)	
Academic shift	Morning	39 (20.1)	24 (20.5)	0.92
	Evening	38 (18.6)	20 (17.9)	
	Night	119 (61.3)	73 (62.4)	

Significant values are in bold (*p* < 0.05). Q1, questionnaire first application; Q2, questionnaire second application.

^†^ Chi-square goodness-of-fit test.

Table 2 shows the reliability analysis (temporal stability and internal consistency) of the DEBQ. We identified Spearman's correlation coefficient ranging from 0.44 (Item 33) to 0.84 (Item 31). Next, based on the original dimensions of the DEBQ (Q1 vs. Q2), we identified a correlation coefficient of 0.82 for the emotional eating score, 0.89 for restrained eating and 0.70 for external eating (data not shown). In addition, Cronbach's alpha coefficient for DEBQ items ranged from 0.91 to 0.92.

**Table 2:** Reliability of the Dutch Eating Behavior Questionnaire (DEBQ).

DEBQ	Q1 score	Q2 score	rho (*p* value)	alpha
Item 1	3 (1 to 3)	2 (1 to 3)	0.72 (<0.001)	0.92
Item 2	3 (3 to 4)	3 (3 to 4)	0.61 (<0.001)	0.92
Item 3	3 (3 to 4)	2 (2 to 3)	0.65 (<0.001)	0.92
Item 4	3 (2 to 4)	3 (2 to 4)	0.71 (<0.001)	0.92
Item 5	3 (1 to 4)	2 (1 to 3)	0.69 (<0.001)	0.91
Item 6	3 (3 to 4)	3 (3 to 4)	0.48 (<0.001)	0.92
Item 7	3 (1 to 4)	3 (1 to 3)	0.74 (<0.001)	0.92
Item 8	3 (1 to 3)	3 (2 to 3)	0.62 (<0.001)	0.91
Item 9	4 (3 to 4)	3 (3 to 4)	0.49 (<0.001)	0.92
Item 10	2 (1 to 3)	2 (1 to 3)	0.67 (<0.001)	0.92
Item 11	3 (1 to 3)	3 (2 to 3)	0.64 (<0.001)	0.92
Item 12	3 (3 to 4)	3 (3 to 4)	0.48 (<0.001)	0.92
Item 13	2 (1 to 3)	2 (1 to 3)	0.74 (<0.001)	0.92
Item 14	2 (1 to 3)	2 (2 to 3)	0.59 (<0.001)	0.92
Item 15	3 (2 to 4)	3 (2 to 3)	0.52 (<0.001)	0.92
Item 16	2 (1 to 3)	2 (1 to 3)	0.57 (<0.001)	0.91
Item 17	3 (2 to 3)	3 (2 to 3)	0.52 (<0.001)	0.92
Item 18	3 (2 to 3)	3 (2 to 3)	0.48 (<0.001)	0.92
Item 19	3 (1 to 3)	3 (2 to 3)	0.72 (<0.001)	0.92
Item 20	3 (1 to 3)	2 (1 to 3)	0.60 (<0.001)	0.91
Item 21	3 (2 to 4)	3 (2 to 3)	0.47 (<0.001)	0.92
Item 22	3 (1 to 3)	3 (1 to 3)	0.75 (<0.001)	0.92
Item 23	2 (1 to 3)	2 (1 to 3)	0.65 (<0.001)	0.91
Item 24	3 (2 to 4)	3 (1 to 4)	0.54 (<0.001)	0.92
Item 25	2 (1 to 3)	2 (1 to 3)	0.76 (<0.001)	0.92
Item 26	2 (1 to 3)	2 (1 to 3)	0.73 (<0.001)	0.91
Item 27	2 (1 to 3)	2 (1 to 3)	0.49 (<0.001)	0.92
Item 28	2 (1 to 3)	2 (1 to 3)	0.67 (<0.001)	0.91
Item 29	2 (1 to 3)	2 (1 to 3)	0.71 (<0.001)	0.92
Item 30	1 (1 to 2)	2 (1 to 2)	0.60 (<0.001)	0.91
Item 31	2 (1 to 3)	3 (1 to 3)	0.84 (<0.001)	0.92
Item 32	2 (1 to 3)	2 (1 to 3)	0.56 (<0.001)	0.91
Item 33	3 (3 to 4)	3 (2 to 4)	0.44 (<0.001)	0.92

Values are median (25th to 75th percentile). Alpha, Cronbach’s alpha coefficient; rho, Spearman correlation coefficient.

Table 3 shows the construct validity (exploratory factor analysis) of the DEBQ. After the data were factored into the exploratory factor analysis (KMO = 0.904; Bartlett's test, *p* < 0.001), we identified a 6-factor structure for the DEBQ. Of these, three factors were the original (with small variations in factors 1 and 3): Factor 1, emotional eating (Items 1, 3, 5, 8, 10, 13, 16, 20, 23, 25, 27, 28, 30 and 32); Factor 2, eating restrained (Items 4, 7, 11, 14, 17, 19, 22, 26, 29 and 31); and Factor 3, external eating (Items 2, 3, 9, 12, 15, 24 and 33). We identified three additional factors: Factor 4, impulsive eating (Items 2, 3, 6, 8, 9, 18 and 21); Factor 5, nonimpulsive eating (Items 14, 17 and 21); and Factor 6, eating by visual or sentimental influence (Items 18, 27 and 30). The variance explained for these factors was 69.1%. Finally, for this 6-factor structure, we observed Cronbach's alpha values of 0.96 for factor 1, 0.92 for factor 2, 0.82 for factor 3, 0.78 for factor 4, 0.56 for factor 5 and 0.70 for factor 6 (data not shown).

**Table 3:** Validity analysis (exploratory factor analysis) of the Dutch Eating Behavior Questionnaire (DEBQ).

DEBQ item	Factor 1	Factor 2	Factor 3	Factor 4	Factor 5	Factor 6	Uniqueness	Communality (1-uniqueness) %
Item 1	0.786						0.313	68.6%
Item 2				0.752			0.280	71.7%
Item 3	0.527		0.334	0.438			0.400	60.1%
Item 4		0.794					0.311	31.1%
Item 5	0.835						0.247	75.3%
Item 6				0.734			0.334	66.6%
Item 7		0.838					0.244	75.6%
Item 8	0.734			0.323			0.309	69.1%
Item 9			0.623	0.483			0.336	66.4%
Item 10	0.841						0.226	77.4%
Item 11		0.745					0.378	62.2%
Item 12			0.641				0.427	57.3%
Item 13	0.818						0.272	72.8%
Item 14		0.328			0.708		0.343	65.7%
Item 15			0.825				0.277	72.3%
Item 16	0.795						0.267	73.3%
Item 17		0.355			0.515		0.580	42.0%
Item 18				0.330		0.666	0.355	64.5%
Item 19		0.841					0.266	73.4%
Item 20	0.825						0.270	73.1%
Item 21				-0.325	0.635		0.439	56.1%
Item 22		0.861					0.212	78.8%
Item 23	0.897						0.140	86.0%
Item 24			0.840				0.183	81.7%
Item 25	0.784						0.267	73.4%
Item 26		0.817					0.266	73.4%
Item 27	0.413					0.688	0.281	71.9%
Item 28	0.880						0.177	82.3%
Item 29		0.787					0.275	72.5%
Item 30	0.732					0.406	0.241	76.0%
Item 31		0.795					0.289	71.1%
Item 32	0.785						0.284	71.6%
Item 33			0.432				0.669	33.1%
***Eigenvalue* (explained variance)**	10.89 (0.33)	5.91 (0.18)	2.51 (0.08)	1.29 (0.04)	1.14 (0.03)	1.05 (0.03)		
**Cumulative explained variance** ^†^	0.691 or 69.1%							

Factor loading < 0.30 was not shown.

^†^ Based on six factors identified by using eigenvalues greater than one rule (Kaiser’s rule).

Table 4 shows the construct validity (confirmatory factor analysis) of the DEBQ. In the SEM, we observed better adjustment for the 6-factor model (based on exploratory analysis), with TLI of 0.87, CFI 0.88, RMSEA of 0.076 and SRMR of 0.073 than for the 3-factor model (based on original DEBQ), with TLI of 0.84, CFI 0.85, RMSEA of 0.083 and SRMR of 0.086. In this sense, we also observed that the four added factors were statistically significant (*p* < 0.001). In the GSEM, we observed that the six-class model has both the smallest AIC (16990.67) and the smallest BIC (17874.38). The same result was observed in the seven-class model; however, by parsimony, the six-class model offered a less complex solution for DEBQ latent class modeling.

**Table 4:** Validity analysis (confirmatory factor analysis) of the Dutch Eating Behavior Questionnaire (DEBQ).

Model structure adjustment	TLI	CFI	RMSEA	SRMR	LR/X²	AIC	BIC
Structural equation modeling
3 factors (original model)	0.84	0.85	0.083	0.086	132.35 (*p* < 0.001)^†^	16242.54	16576.38
6 factors (EFA model)	0.87	0.88	0.076	0.073		16134.19	16507.31
Generalized structural equation modeling	*n*	df	Log likelihood (full model)			AIC	BIC
3 factors	195	134	−8818.05			17904.09	18342.67
4 factors	195	168	−8574.01			17484.02	18033.89
5 factors	195	202	−8397.00			17198.01	17859.16
6 factors	195	270	−8225.34			16990.67	17874.38
7 factors	195	270	−8225.34			16990.67	17874.38

AIC, Akaike information criterion; BIC, Bayesian information criterion; CFI, comparative fit index; df, degrees of freedom; EFA, exploratory factor analysis. n, number of observations; RMSEA, root mean square error of approximation; SRMR, standardized root mean squared residual; TLI, Tucker–Lewis Index; LR/X², likelihood-ratio test/chi-square.

^†^ Comparing 3-factor with 6-factor model.

## Discussion

During the outbreak of COVID-19, several measures were taken in epidemiology and public health, including the replacement of data collection and face-to-face research by remote data collection (5). The novelty of this study was to test the psychometric properties of a questionnaire to measure the eating behavior construct in university students from low-income regions. The findings of this study showed that the DEBQ (online format) is reliable and valid for measuring this construct remotely. Thus, the questionnaire can be a viable and low-cost tool to study eating behavior under conditions of restricted social contact (as experienced during COVID-19).

Although we observed a significant rejection/refusal rate in face-to-face recruitment (and response to Q1) and in subsequent contacts *via* social network messages (and responses to Q2), there was no differential bias between the two applications. Methodological studies have observed a high prevalence of rejection/refusal in studies conducted in Latin America (26) and observational studies addressing European university students (18) and Brazilians (17). However, the proportion of rejection is higher at lower socioeconomic levels or in lower income regions, in line with the literature (26). In addition, we attributed the high rejection rate (between applications Q1 and Q2) to the reduced motivation of participants to complete a second questionnaire in a short time (two-week interval). In this regard, recent studies also indicate that socioeconomic and educational conditions may be a limiting factor for access to and adherence to online research in a pandemic context (4, 5). Thus, we recommend that future studies in low-income regions, with applications of online tools, influence the size of the designed sample (26) (especially for male participants aged 26 to 35 years old) and that conduct combined recruitment and participation approaches (e.g., adopt *snowball* sampling, allow participation *via* telephone of a relative or colleague, simultaneous reminders *via* social network, SMS and face-to-face, among others) (5, 27).

Our findings indicated reliability and acceptable validity for the online version of the DEBQ in low-income university students. These findings corroborate the versions adapted to European adults (7, 8, 10, 28) and university students (6–9) in the face-to-face and online format. All previous questionnaires showed a 3-factor structure. In this sense, we identified the three original factors of DEBQ, emotional eating (with the inclusion of Item 27), restrained eating and external eating (with the inclusion of Item 3 and absence of Items 6 and 27), and three additional factors (labeled impulsive eating, nonimpulsive eating and eating by visual or sentimental influence). The additional factors were mainly grouped by items of the original composition of emotional eating and external eating. However, in terms of global fit values, the 6-factor solution presented a better structure than the original 3-factor solution in low-income university students during the pandemic. There is no clear evidence of the influence or changes caused by the lockdown, social isolation and uncertainty in eating behavior, although adherence to healthy eating habits throughout the COVID-19 pandemic seems to have worsened (2). However, we speculate that external stimuli *via* traditional media and social networks facilitated access to enticing foods (of high caloric value) *via* delivery applications and that the restriction of social contact may cause an imbalance in the mechanisms of emotional eating, since it is more related to psychological than nutritional aspects, revealing latent factors of DEBQ in a pandemic context. Thus, this finding suggests that the social detachment resulting from the pandemic exposed the university students to an abrupt change in the environment that may have an effect, even if transitory, on the behavior construct studied (4).

Some limitations of this study should be mentioned. Although our sample was based on diverse parameters (age range, biological sex and academic course) consistent with previous studies (17, 18), the sample design (size and recruitment) does not allow extrapolation of the results of this study beyond the psychometric findings. In this sense, we observed a high rate of rejection/refusal. However, in *post hoc* analysis, the power of the sample (for the lowest correlation observed = 0.44, Item 33, Table 2) was 99.0% (*β* = 0.01, one-tailed), surpassing the power adopted in the design of the sample size of 90.0%. Additionally, the research site was selected by convenience, and the sampling process was stratified because the economic and logistical costs of a representative sample would make a methodological study unfeasible (26), in addition to the ethical precepts involved in increasing the recruitment of participants without clear scientific gain (15). Thus, the demographic, economic and academic characteristics present in the chosen institution were sufficient to reproduce the sample diversity present in university students from low-income regions of Brazil (17). Finally, the level of education of the participants may indicate that these university students have a better understanding of the questionnaire, which may result in more accurate responses and distance them from their nonuniversity peers. Further work is needed to evaluate whether this more detailed factor structure adds to DEBQ's ability to identify eating behavior domains during a pandemic *via* online formats in low-income regions.

## Conclusion

The *online* format of the *Dutch Eating Behavior Questionnaire* presents acceptable reliability and validity for measuring the eating behavior construct in university students from low-income regions. The questionnaire is a viable and low-cost alternative for application in conditions of social detachment, such as during a pandemic.

## Data Availability

The raw data supporting the conclusions of this article will be made available by the authors, without undue reservation.
